# Tail Flick Modification of Orexin-A Induced Changes of Electrophysiological Parameters in The Rostral Ventromedial Medulla

**Published:** 2014-05-25

**Authors:** Hassan Azhdari-Zarmehri, Saeed Semnanian, Yaghoub Fathollahi, Firouz Ghaderi Pakdel

**Affiliations:** 1 Department of Physiology, School of Medical Sciences, Tarbiat Modares University, Tehran, Iran; 2 Department of Basic Sciences, Torbat Heydarieh University of Medical Sciences, Torbat Heydarieh, Iran; 3 Department of Physiology, Faculty of Medicine, Urmia University of Medical Sciences, Urmia, Iran

**Keywords:** Orexin-A, Tail Flick, Pain

## Abstract

**Objective:**

It is well known that intracerebroventricular (ICV) and supraspinal injections of orexin-A elicit analgesia, but the mechanism(s) of action remains unidentified. This study aims to characterize the effect of orexin-A on rostral ventromedial medulla (RVM) neurons which are involved in the descending nociception modulating pathway.

**Materials and Methods:**

In this experimental study, we injected orexin-A or vehicle di- rectly into rats’ ICV while the tail flick (TF) latencies were measured and the on- and off-cell firing activities were monitored for more than 60 minutes.

**Results:**

In response to noxious stimuli on the tail, we observed increased firing rate of on-cells and a decreased association with the firing rate of off-cells and in neutral cells the firing rate was unchanged just prior to tail flicking. ICV injection of orexin-A decreased the spontaneous firing rate of on-cells (the type of RVM neurons believed to have facilitatory action on nociception). Furthermore, orexin-A increased firing rate of off-cells (the type of RVM neurons believed to have an inhibitory action on nocicep- tion). Orexin-A reduced the TF-related responses of on-cells and TF-related pause duration of off-cells.

**Conclusion:**

These results have shown that the analgesic effect produced by orexin-A may be induced by brainstem neurons. It is suggested that the orexinergic system from the hypothalamus to the RVM may have a potential role in modulation of nociceptive transmission.

## Introduction

The neuropeptide orexin (hypocretin) has been simultaneously discovered by two independent research teams (1, 2). The orexin-containing neurons are located exclusively within the lateral hypothalamus (2, 3), where they are believed to be involved in pain modulation through the brain stem (4, 5). Sakurai et al. (2) have also described two orexin receptors coupled to G proteins. These receptor subtypes are differentially localized in the central nervous system, particularly in nuclei involved in pain processing (6-8). Widespread projections of orexin have been implicated in the regulation of various brain and body functions such as feeding (2), sleep (9, 10), stress-induced analgesia (11, 12), reward and addiction (13-15). Recently, it has been proposed that systemic injection of orexin-A is involved in pain modulation through orexin receptor 1 (16-19). A recent study has reported an association with orexin-A induced analgesia at the level of the supraspinal and spinal cord (20-22). A finding that intra-periaqueductal gray matter (*PAG*) microinjection of orexin-A has an analgesic effect in hot-plate and formalin tests in rats confirms that *PAG*, as a supraspinal center, is involved in orexin-A induced analgesia through the orexin receptor 1 (20, 22). Additionally, rostral ventromedial medulla (RVM) is involved in the top-down pain-modulating system through the first synapse in dorsal horn neurons (23) and innervated by orexinergic fibers (3). Thus, RVM may directly and/or indirectly (projection from *PAG*) be an important site of action for orexin-induced supraspinal antinociception. As described by Fields and Heinricher (24), RVM neural response to noxious stimulus applied on the tail or paw can be divided into three distinct physiological groups: off-, on-and neutral-cell.

Although the effect of orexin-A on RVM neurons is unclear, the responses these neurons to opioid induced- analgesia are well known (23, 25). Therefore this study researches the effects of intracerebroventricular (ICV) injection of orexin-A on RVM neuronal activity in rats.

## Materials and Methods

In this experimental study, all experiments were performed in accordance with the National Institutes of Health Guide for the Care and Use of Laboratory Animals (NIH Publication No. 80-23, revised 1996) and approved by the Ethics Committee of the School of Medical Sciences, Tarbiat Modares University (TMU), Tehran, Iran. Adult Sprague-Dawley rats (220-300 g at the beginning of the experiments) were purchased from Razi Institute (Hesarak Karaj, Iran). Animals were randomly housed in groups of three rats per cage in a temperature-controlled room under a 12 hour light-dark cycle with lights on from 7:00 to 19:00. Animals had access to food and water ad libitum. For all experiments, we complied with the regulations of local authorities for handling laboratory animals.

### Surgical procedure

The procedure for simultaneous extracellular single unit recording and noxious response was adopted from previous studies (23, 26). Animals were anesthetized with sodium pentobarbital (60 mg/kg i.p., Sigma-Aldrich, USA) and additional doses (10-15 mg/kg/h, iv) were administered through a catheter that was inserted into an external jugular vein with the intent to maintain a level of light anesthesia (27, 28). Tracheal cannulation was performed for spontaneous ventilation after which the animals were placed into a stereotaxic instrument (29, 30). Using a thermistor-controlled heating pad (Bahrami Thermostat Pad, Kerman, Iran), body temperature was maintained at 35.5-36.8˚C. A guide cannula for ICV injections (23-gauge) was implanted into a region 2 mm above the right lateral ventricle using the following stereotaxic coordinates: 0.96 mm caudal to bregma, 2 mm laterally from midline, and 1.8 mm ventrally from the level of the bregma. The ICV cannula was anchored on the skull with a stainless steel screw using dental cement. A 2 mm hole was drilled in the skull above the RVM at the following stereotaxic coordinates (31), 10.68-11.28 mm posterior to the bregma, in midline and the dura was reflected to allow insertion of a recording electrode. Following surgery, the animals were maintained in a lightly anesthetized state using a continuous infusion of sodium pentobarbital (10-15 mg/kg/h, iv) as previously described (27, 28).

### Tail flick testing

The tail flick (TF) latency was used as a measure of nociceptive response. Each trial consisted of a linear increase in temperature at 1.8˚C/s from a holding temperature of 34˚C until the TF occurred or to a maximum of 52˚C at less than 10 seconds. Trials were carried out at 5-minute intervals throughout the experiment (32).

### Extracellular single unit recording

Extracellular recordings from individual neurons were obtained with glass micropipettes (2-5 MΩ impedance) filled with 2% pontamine sky blue dye in 0.5 M sodium acetate. Micropipettes were stereotaxically advanced into the RVM (10-10.5 mm ventral to the skull surface). Micropipette recordings were amplified by a microelectrode amplifier (DAM 80, WPI), displayed continuously on a storage oscilloscope as unfiltered and filtered (300 Hz-3 kHz bandpass) signals, and monitored with an audio monitor (WPI, USA). Spike waveforms were monitored and stored on a personal computer (PC) with cool edit and Igor pro (WaveMetrics, Lake Oswego, OR) software for off-line analysis to ensure the unit under study was unambiguously discriminated throughout the experiment. The RVM neurons were classified as previously described (23, 24). Off-cells were characterized by a pause in spontaneous activity just prior to the TF. On-cells were characterized by a burst of spontaneous activity before the TF. Neutral-cells were characterized by no change in spontaneous activity associated with TF and no response to noxious stimulation.

### Intracerebroventricular (ICV) microinjection

Rats were stabilized with light anesthesia. Next, a 30 gauge microinjection cannula that had a tip 2 mm greater than the guide cannula was connected to a 10 μl Hamilton syringe and inserted through the guide cannula. We performed three nociceptive testing trials and orexin-A (20 μM, 5 μl, Sigma, USA) was injected over a 2-minute period.

### Protocol and data analysis

We determined the effect of orexin-A microinjection into the ICV on TF latency and on the ongoing and reflex-related discharges of RVM neurons. Following three baseline TF trials, either orexin-A or savehicle were infused into the ICV. Orexin-A was dissolved in saline and the aliquots were stored at -20˚C until use. All ICV injections were made in a volume of 5 μl , aliquots had saline added to reach a volume of 5 microliters and injected over a period of 2 minutes. TF latency and cell activity were monitored for an additional period of 60 minutes (32).

Only one protocol was performed in each rat. In two cases, two on-cells were recorded on a single electrode. The average TF latencies and cell parameters were calculated at the baseline period and compared with 3 trials after the drug microinjection.

Three parameters of cells were analyzed (32, 33) as follows: 1. baseline spontaneous neural activity; 2. ongoing activity where we used the 30 second period prior to each TF trial as an overall index of ongoing firing; and 3. frequency analysis for each neuron. For frequency analysis, we computed the firing rate over 500 ms epochs before and after orexin-A administration after which the percentage of the time in the same firing rate was plotted.

After a unit was isolated and its stability determined, the pre-injection spontaneous firing rate was calculated. Then orexin-A or saline were microinjected into the ICV and the effect of their administration on the unit activity of RVM neurons was observed.

### Histology

**Fig 1:**
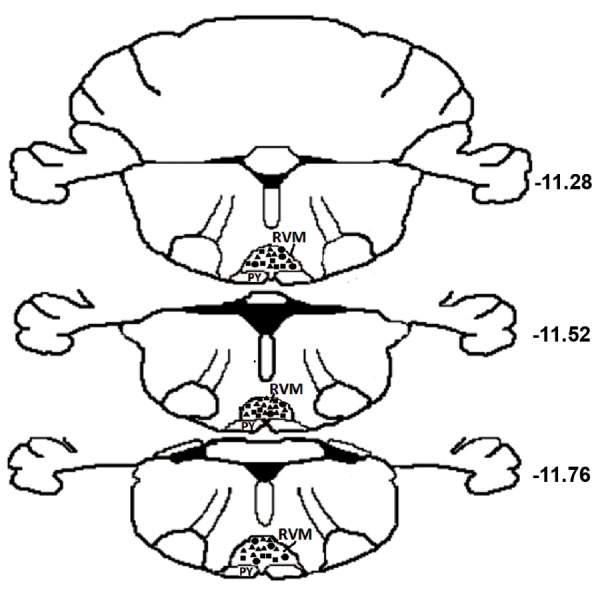
Location of recording sites in the rostral ventromedial medulla (RVM). The plane was modified to a series of three sections for the brain areas according to the atlas of Paxinos and Watson (31). Representative coronal sections show histologically verified locations of 24 on-cells (black triangles), 18 off-cells (black square) and 10 neutral-cells (black circle). Numbers to the right of each section indicate the distance from the caudal to the bregma.

The tips of the micropipettes were marked by iontophoretic ejection of pontamine sky blue with negative current pulses (10 minutes) in the RVM (21, 22). At the end of the experiments, rats were deeply anesthetized with a urethane overdose (1.2 g/kg); a volume of 5 μl of pontamine sky blue (0.2%) was also injected ICV at 10–20 minutes before the animals were sacrificed. Rats were then perfused intracardially with 100 ml of 4% formalin after which the brains were removed and sectioned. Coronal slices of 200-300 μm thicknesses were then cut through the brain stem from the trapezoid body to the inferior olivary nuclei using a vibrating microtome (Vibatome 1000 plus, US). Locations of recording sites were identified according to the atlas of Paxinos and Watson (31). The RVM was comprised of the nucleus raphe magnus and nucleus reticularis gigantocellularis pars alpha (Gia) (Fig 1).

### Statistical analysis

The obtained results are expressed as means ± standard error of mean (SEM). The data were subjected to the paired t test, one-way or twoway analysis of variance (ANOVA) which were followed by protected Dunnett’s test for multiple comparisons, as needed. P<0.05 was considered to be significant.

## Results

**Fig 2:**
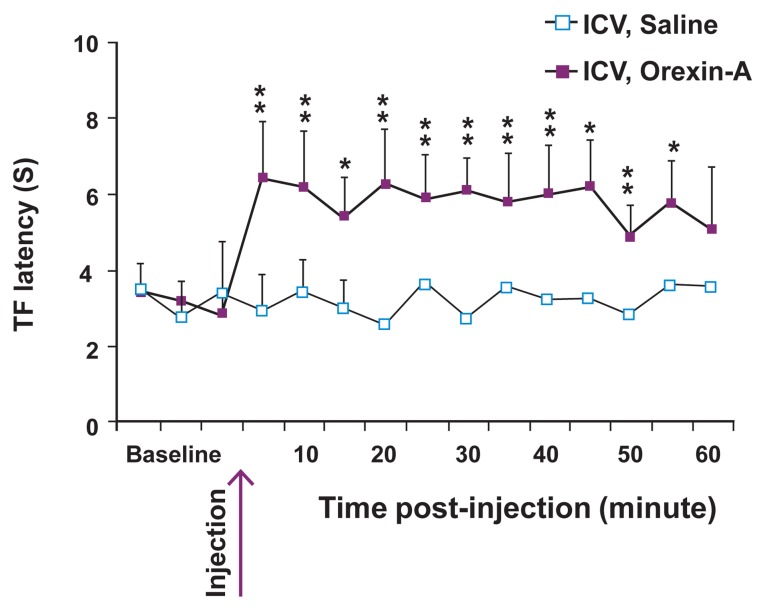
Intracerebroventricular (ICV) microinjection of orexin- A (20 μM) produced thermal analgesia. Orexin-A produced a significant increase in tail flick (TF) latency when microinjected into the right ventricle compared to vehicle (saline, 5 μl). There was no difference among the groups in baseline latencies. Data were successfully recorded in 40 animals in the orexin-A group and 13 out of 13 animals in the vehicle group.

**Fig 3:**
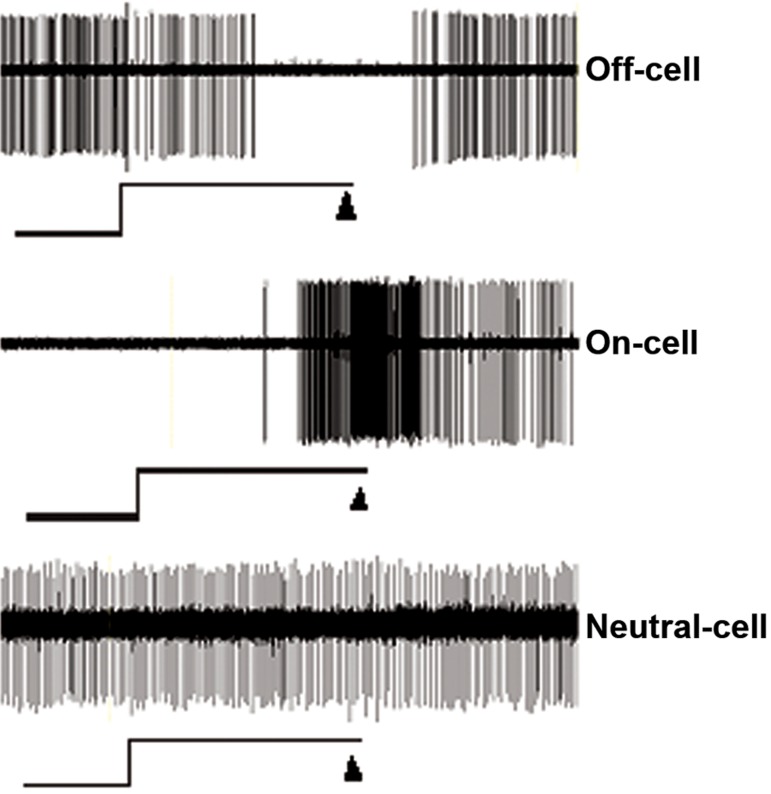
Single oscilloscope sweep shows activity of a representative off, on and neutral-cells during the tail heat trial. Top trace is cell discharge, the middle line shows heat exposure and stop, and the lower triangles show tail withdrawal point.

Data analysis according to two-way ANOVA followed by protected Dunnett’s test showed that ICV application of orexin-A (20 μM) in lightly anesthetized rats produced an increase in TF latency whereas infusion of saline (vehicle) into the ICV had no effect on TF latency (Fig 2). We recorded 42 neurons from 40 animals in the orexin-A group and 13 neurons from 13 animals in the vehicle group. In this study, we discriminated three physiological types of RVM neurons according to a previous study (32). The off-cells and on-cells showed a pause and sudden increase in activity just before tail withdrawal reflexes, respectively . Activity of the neutral cells remained unchanged corresponding with the tail withdrawal reflexes (Fig 3).

**Fig 4:**
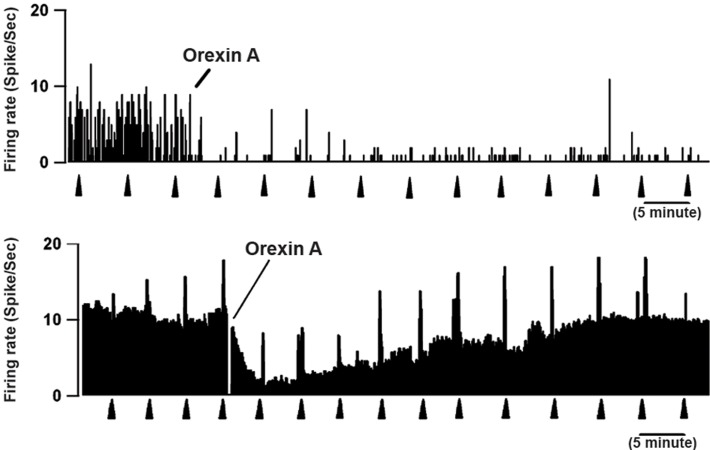
Rate-meter records show the effect of intracerebroventricular (ICV) orexin-A injection on spontaneous firing rate and ongoing discharge of 2 on-cells in the rostral ventromedial medulla (RVM). In both cases, firing pattern and rate decreased by orexin administration. Triangles indicate tail flick (TF) trials. 1-s bins.

**Fig 5:**
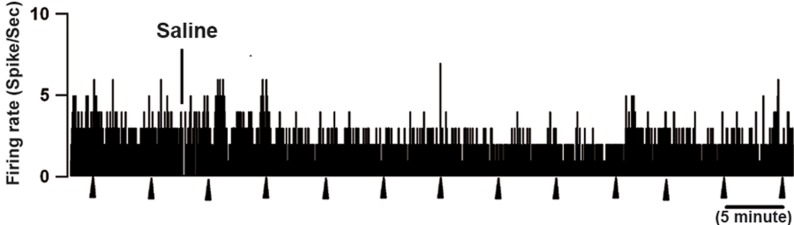
Vehicle infusion showed no effect on on-cell discharge. On-cells were recorded during infusion of saline vehicle in the ventricle. Firing pattern and rate were unaltered. Triangles indicate tail flick (TF) trials. 1-s bins.

After the stability of extracellular single unit recording from the RVM neurons, orexin-A (20 μM, 5 μl) was injected into the right ICV of the rats. We successfully recorded a total of 21 on-cells, 15 off-cells and 6 neutral-cells in RVM. Administration of orexin- A inhibited or decreased spontaneous basal activity, which was 64.57 ± 5% at baseline and decreased to 42.13 ± 5.94% (p<0.001 in 13 of 21 on-cells). Following ICV microinjection of orexin-A, 13 out of 21 on-cells displayed a decrease in ongoing activity (paired t test; p<0.05, Fig 4). On the other hand, as demonstrated in figure 5, the vehicle microinjection did not affect spontaneous firing rate and firing activity associated with reflex-related response which was 4.46 ± 2.82 sp/s at baseline and 4.29 ± 3.63 sp/s after saline injection (n=5, paired t test, p>0.05, Fig 5).

**Fig 6:**
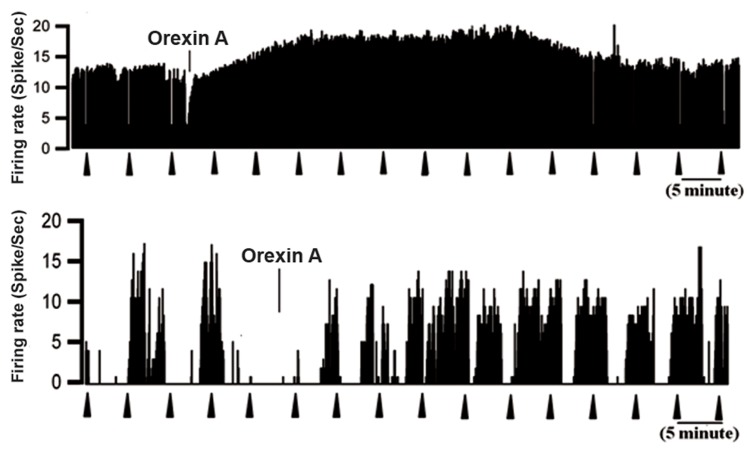
Rate-meter records show effects of ICV injection of orexin-A on spontaneous activity and ongoing discharge of identified off-cells in the RVM. Top: excitation of an offcell after ICV injection of orexin-A. Bottom: This neuron showed substantial cycling activity throughout recording, but active periods were increased following orexin-A injection. Triangles indicate TF trials. 1-s bins.

**Fig 7:**
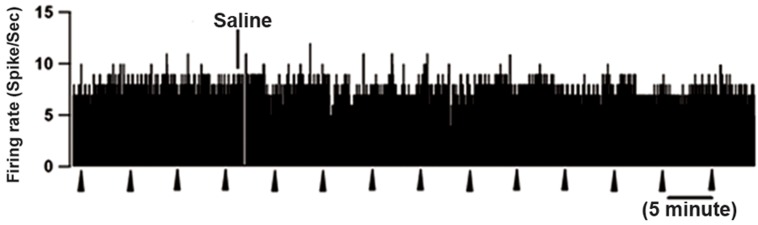
Vehicle infusion had no effect on off-cell discharge. Off-cells were recorded during infusion of saline in the ventricle. Firing pattern and rate were unaltered. Top three histograms of each cell show point of TF (black triangle) and stimulus-related changes in firing rate before (a) and after (b and c in different time) ICV saline injection. Triangles indicate TF trials. 1-s bins.

**Fig 8:**
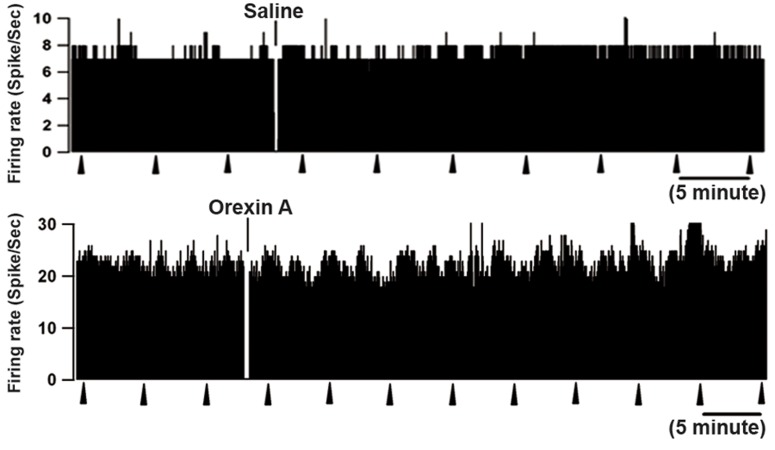
Rate-meter records show lack of effect of ICV saline and orexin-A injection on the ongoing discharge of 2 neutral- cells in the RVM. In both cases, firing pattern and rate were completely unaffected by orexin. Triangles indicate TF trials. 1-s bins.

**Fig 9:**
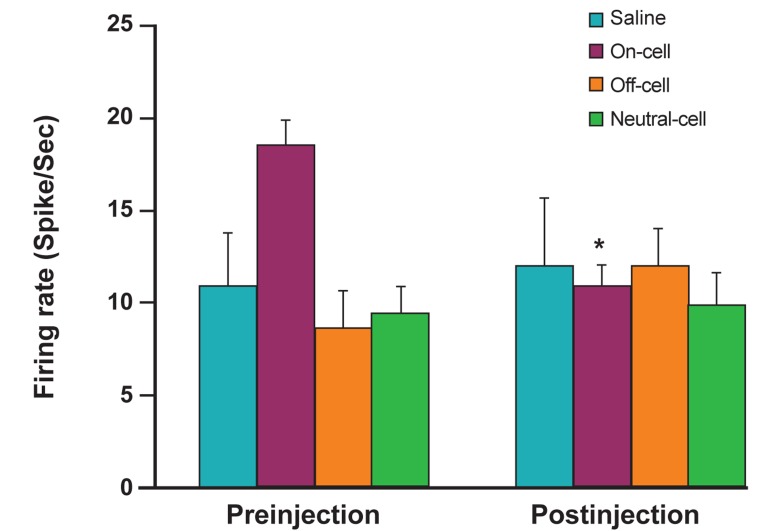
Mean ongoing firing of on-cells, off-cells, and neutral cells at baseline compared with the post-injection period in animals that received intracerebroventricular (ICV) microinjections of orexin-A or vehicle. *p<0.05, discharges following orexin-A compared with the value at baseline.

**Fig 10:**
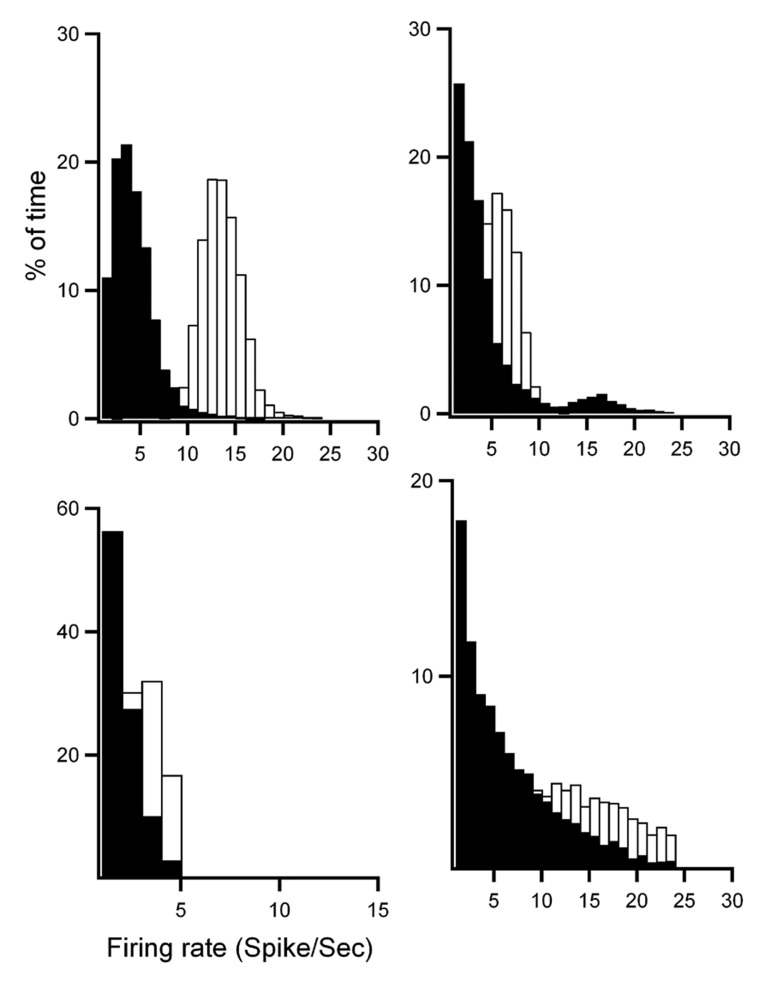
Frequency analysis of firing rate distributions of four on-cells in the rostral ventromedial medulla (RVM) at baseline (white bars) and after orexin-A injection (black bars). Firing rate was measured for each bin (500 ms) and plotted as a percentage of baseline activity and after orexin-A injection for each cell. Orexin-A induced a shift to the left of the firing rate of on-cells indicating a probability decrease in high firing rate after orexin-A injection.

**Fig 11:**
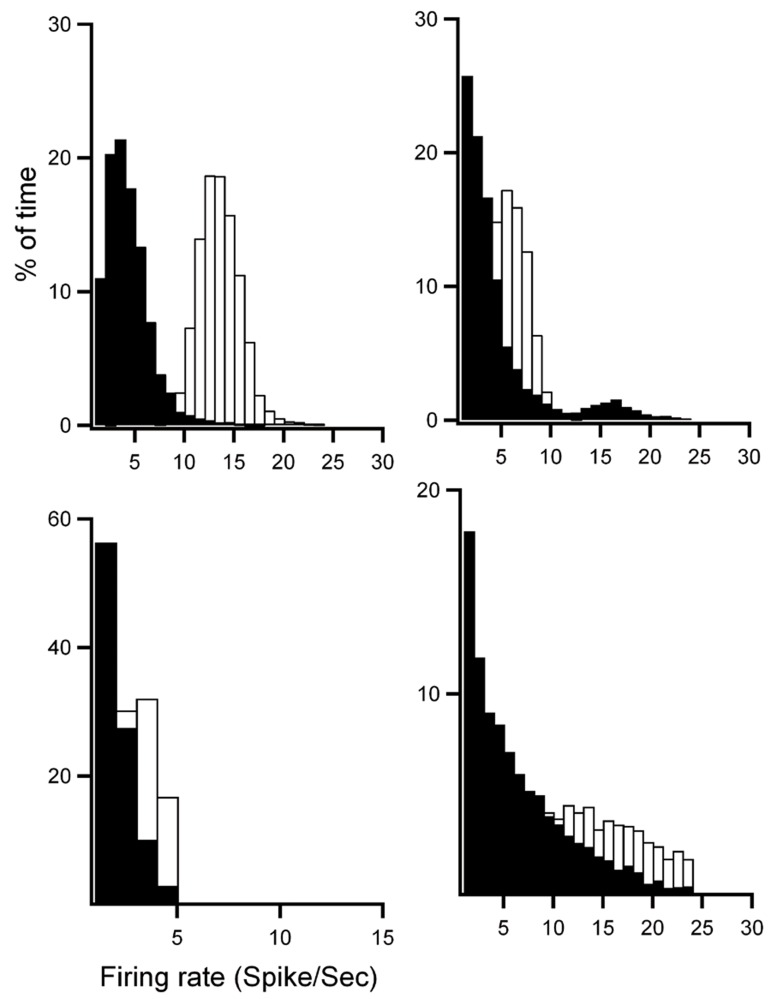
Frequency analysis of firing rate distributions of four off-cells of rostral ventromedial medulla (RVM) in baseline (white bars) and after orexin-A injection (black bars). Firing rate was measured for each bin (500 ms) and plotted as a percent of time in baseline and after orexin-A injection for each cell. Orexin-A induced a shift to the right of the firing rate indicating an increase in frequency after the injection.

As seen in Figure 6, the off-cells had an increased spontaneous firing rate from 6.74 ± 1.44 sp/s at baseline to 9.34 ± 0.65 sp/s after administration of orexin- A (p<0.05, Fig 6). Vehicle microinjection had no effect on off-cell firing rate (n=4, Fig 7). As shown in figure 8, the firing rate and ongoing activity of neutral-cells remained unchanged following ICV injection of saline and orexin-A (Fig 8, p>0.05). Additionally, there was an increase in ongoing activity following ICV microinjection of orexin-A, but it was not statistically significant (paired t test, Fig 9). Frequency analysis of the firing rate distributions of the four on-cells in RVM in baseline (white bars) and after Orexin-A injection (black bars) showed that Orexin-A induced a shift to the left of the firing rate of on-cells, indicating a probability decrease in low firing rate after Orexin- A injection (p<0.01, Fig 10). Frequency analysis of the firing rate of the four off-cells in RVM at baseline (white bars) and after orexin-A injection (black bars) showed that orexin-A induced a shift to the right of the firing rate of off-cells which indicated an increased probability in high firing rate after orexin-A injection (paired t test, p<0.01, Fig 11).

## Discussion

Orexinergic neurons have widespread projections from the lateral hypothalamus that lead to their involvement in a number of different functions, including feeding (2), sleep (9, 10), stress induced analgesia (11, 12), reward and addiction (13-15). More recently, the role of orexin in pain modulation has been demonstrated (16, 17, 20, 22, 34, 35). In a study, central administration of orexin-A produced a dose-dependent antinociceptive effect in the hotplate test model in rats . In other mouse models, orexin-A inhibited visceral nociception and thermal hyperalgesia induced by an intraplantar carrageenan injection model produced an efficacy equivalent to morphine (17). Morphological studies have established that the orexin-containing neurons are distributed along most parts of pain circuitry including the RVM region which is considered important for pain modulation (3).

Although most studies are concerned with orexin-A induced analgesia through the spinal cord but, specific brain regions that include *PAG* and paragigantocellularis lateralis have been implicated in the antinociceptive effect of orexin-A (21, 22). We have previously shown that infusion of orexin-A into the RVM caused a decrease in formalin-induced nociceptive behaviors (36). It has been determined that intra- *PAG* microinjection of orexin-A elicits an analgesic effect in the hot-plate and formalin tests for rats, which confirms that *PAG* is a supraspinal center involved in orexin-A induced analgesia through orexin receptor 1 (20,22). The principal finding of the present study h as shown that ICV administration of orexin-A inhibited the spontaneous firing rate and ongoing activity of on-cells while it produced thermal analgesia. In addition, orexin-A activated the spontaneous firing rate of off-cells whereas the neutral-cells were unaffected. These data supported previous behavioral observations 20, 22 in which orexin-A produced an analgesic effect. The decrease in on-cell firing rate by orexin-A and increase in off-cell spontaneous firing rate might be involved in orexin-A induced analgesia. Thus, our findings suggested that the RVM might either directly or indirectly be necessary for an orexin-A induced analgesic effect.

Two reports have emphasized the presence of orexin-induced analgesia through a top-down modulating system (20, 22). Production of orexin is restricted to the lateral hypothalamus (1, 2), a region known to modulate pain processing through the brain stem (4). This has supported the current study’s hypothesis that either direct or indirect action of orexin-A is mediated through RVM neurons. RVM is involved in a top-down pain-modulating system through the first synapse in dorsal horn neurons (23) and innervated by orexinergic fibers (3). Thus, RVM may directly and/or indirectly (projection from *PAG*) be an important site of action for orexin-induced supraspinal antinociception.

We believed that orexin-A might indirectly activate off-cells via disinhibition. In this model, orexin- A activated on-cells as inhibitory interneurons responsible for disinhibition of off-cells; these, in turn, induced antinociception through the dorsal horn neuron in the spinal cord.

Orexin has been shown to produce excitatory effects on most areas in the brain in addition to inhibitory effects on a few neurons obtained from other regions (37, 40). The latter observation supports the indirect effect of orexin-A. Indirect effects of orexin-A have been shown in other brain regions such as the substantia nigra (37), laterodorsal tegmental nucleus (39), hypothalamus (40), and raphe nucleus (38).

In consistent to above studies, the data of the present study speculated that Orexin-A has bidirectional effects on RVM neurons, to recognize the direct or indirect mechanism(s), we might require further studies at the cellular and/or molecular levels.

## Conclusion

These results have shown that orexin-A might produce analgesia through activation/inhibition of RVM neurons required for pain modulation. It has been suggested that the orexinergic system from the hypothalamus to RVM might have a potential role in modulation of nociceptive transmission. Although orexin-A and morphine have similar effects as antinociceptives, these substances have extremely different actions in arousal conditions.
